# What Is the Matter? Or, Report of a Case by Dr. John Coston, White House, Henry County, Georgia

**Published:** 1857-02

**Authors:** 


### ARTICLE V.

*What is the matter? or Report of a case by* Dr. John Coston, *White House, Henry Co., Ga.*

Messers Editors.—It may be thought strange that I should attempt the report of a case under the above caption; but perhaps you may discover before I am done, that it is easier to ask than to answer the question; but my desire is to elicit an answer from you or some of your contributors, and especially as the case is still on hand, and if any thing can be done for the relief of the patient, I am anxious to be directed in the proper course. Milton G., aged 5 years, strumous constitution, but well grown and apparently healthy up to last April; about that time exhibited the evidences of disease, by fretfulness and voracity of appetite, which continued to increase, with the addition of other abnormal manifestations until July, when I accidentally saw him while attending another member of the family. Upon examination, I found him in the following condition: his eyes, which were formerly straight, now squinted badly, and presented an idiotic expression; his speech formerly quick, was now hesitating, so much so as to require as much time to articulate a monosylable as formerly to speak several words; his head enlarged generally, with a puffy appearance, though quite firm to the touch. In addition to the above, his appetite continued to be insatiable.

On the 2d of August, I was requested to see him regularly, and found the symptoms greatly aggravated; the blood-vessels of the head were greatly distended, and appeared as if the blood was coagulated in them, resembling dark cords under the integument.

August 14th—No improvement in his symptoms and becoming very restless. I applied a blister to the back of the neck, which had the effect of quieting him considerably, and reducing the puffed condition of the head to some extent; he continued in about the same condition until the 19th of August, when he was found to be somewhat Amaurotic, and on the 22d, was evidently laboring under complete Amaurosis; from this period, up to the 28th September, there was no material change in his condition, about which date his appetite failed for the first time, and after this he ate but little.

About this period, some spasmodic action manifested itself, affecting principally the extremities; he gradually became very much emaciated, and about the 15th of October he lost the power of speech and hearing, which continued for about 8 days, when the speech, hearing and appetite returned; in a few days, however, he became exceedingly restless, and continued almost constantly to cry out for food: his limbs now became rigidly flexed, his left hand was drawn up near the chin, his wrist drawn inwardly, thumb drawn across the palm of the hand, and fingers tightly clenched; his right arm was drawn in a somewhat similar way, though not to the same extent, some little use of the hand being retained; his left thigh was drawn upon the abdomen, and the leg was flexed upon the thigh; the ankle was drawn inwards and downwards; the toes were drawn entirely under the feet; the right leg and foot were also in a somewhat similar condition, though not to the same extent. With occasional changes of a slight character, he has continued in about the same condition, up to the present time.

You will perceive that the condition of the stomach, bowels, and urinary organs has not been referred to, and for the reason that no important deviation from the state of health was observed, farther than the morbid appetite may have had connection with an abnormal condition of the stomach.

You will also perceive that I have said nothing of the treatment instituted, save the application of a blister to the nape of the neck, and for the reason, that this was the only remedy that seemed to have the slightest influence over the course of the disease, and on this account might possibly throw some light on the true pathology of the case.

